# Prion-Like Seeding of Misfolded α-Synuclein in the Brains of Dementia with Lewy Body Patients in RT-QUIC

**DOI:** 10.1007/s12035-017-0624-1

**Published:** 2017-05-26

**Authors:** Kazunori Sano, Ryuichiro Atarashi, Katsuya Satoh, Daisuke Ishibashi, Takehiro Nakagaki, Yasushi Iwasaki, Mari Yoshida, Shigeo Murayama, Kenichi Mishima, Noriyuki Nishida

**Affiliations:** 1 Department of Physiology and Pharmacology, Faculty of Pharmaceutical Sciences, Fukuoka University, 8-19-1 Nanakuma, Jonan-ku, Fukuoka, 814-0180 Japan; 2 Division of Microbiology, Department of Infectious Diseases, Faculty of Medicine, University of Miyazaki, Miyazaki, 889-1692 Japan; 3 Department of Locomotive Rehabilitation Science, Nagasaki University Graduate School of Biomedical Sciences, Nagasaki, 852-8523 Japan; 4 Department of Molecular Microbiology and Immunology, Nagasaki University Graduate School of Biomedical Sciences, Nagasaki, 852-8523 Japan; 5 Department of Neuropathology, Institute for Medical Science of Aging, Aichi Medical University, Aichi, 480-1195 Japan; 6 Department of Neuropathology, Tokyo Metropolitan Geriatric Hospital and Institute of Gerontology, Tokyo, 173-0015 Japan

**Keywords:** α-synuclein, Real-time quaking-induced conversion (RT-QUIC), Prion, Dementia with Lewy bodies (DLB)

## Abstract

The prion-like seeding of misfolded α-synuclein (αSyn) involved in the pathogenesis of Lewy body diseases (LBD) remains poorly understood at the molecular level. Using the real-time quaking-induced conversion (RT-QUIC) seeding assay, we investigated whether brain tissues from cases of dementia with Lewy bodies (DLB), which contain serine 129 (Ser129)-phosphorylated insoluble aggregates of αSyn, can convert *Escherichia coli*-derived recombinant αSyn (r-αSyn) to fibrils. Diffuse neocortical DLB yielded 50% seeding dose (SD_50_) values of 10^7^~10^10^/g brain. Limbic DLB was estimated to have an SD_50_ value of ~10^5^/g brain. Furthermore, RT-QUIC assay discriminated DLB from other neurological and neurodegenerative disorders. Unexpectedly, the prion-like seeding was reconstructed in reactions seeded with oligomer-like species, but not with insoluble aggregates of r-αSyn, regardless of Ser129 phosphorylation status. Our findings suggest that RT-QUIC using r-αSyn can be applied to detect seeding activity in LBD, and the culprit that causes prion-like seeding may be oligomeric forms of αSyn.

**Electronic supplementary material:**

The online version of this article (doi:10.1007/s12035-017-0624-1) contains supplementary material, which is available to authorized users.

## Introduction

The accumulation of abundant misfolded proteins in the brain is a defining feature of most neurodegenerative disorders. Lewy body diseases (LBD), such as dementia with Lewy bodies (DLB) and Parkinson’s disease (PD), are characterized by the presence of Lewy bodies (LB), which are filamentous cytoplasmic inclusions composed mainly of aggregated α-synuclein (αSyn). Although the pathogenic mechanisms have not been fully elucidated, LBD is thought to occur through the accumulation of LB, i.e., aggregated αSyn, in neurons and neurites.

The bulk of αSyn aggregates within LB are phosphorylated at serine 129 (Ser129), while αSyn in the normal brain undergoes very little phosphorylation. Phosphorylation at Ser129 is the dominant pathological characteristic and may be crucial in LB formation and the pathogenesis of LBD. Ser129 phosphorylation accelerates polymerization of recombinant αSyn (r-αSyn) [1], and overexpression of wild-type (WT) r-αSyn, but not nonphosphorylatable mutant with substitution of alanine for Ser129 (S129A), induced significant accumulation of LB-like inclusions in cultured cells [2]. Other groups demonstrated that Ser129 phosphorylation inhibits the fibrillation of r-αSyn [3], and mutation of S129A increases inclusion formation in a *Drosophila* model [4]. The study using *Drosophila* also revealed that the number of inclusion bodies is inversely correlated with toxicity to dopaminergic neurons, indicating that inclusion bodies protect against neuronal toxicity [4]. However, studies in rat models using recombinant adeno-associated virus (rAAV) to overexpress WT and mutant αSyn indicated that nonphosphorylated αSyn is associated with more severe toxicity toward dopaminergic neurons than the phosphorylated form [5, 6], and that there are no significant differences in neurotoxicity between both forms [7]. Thus, the significance of Ser129 phosphorylation of αSyn in the pathogenesis of LBD remains poorly understood.

Postmortem studies by Braak and colleagues showed that LB pathologies initially arise in the medulla oblongata and the olfactory bulb and then extend to the midbrain and the limbic areas, with subsequent spread to the neocortical regions in PD [8]. In addition, the pathological stage appears to be closely linked to progression of lesions and clinical manifestations [9]. The spread of LB in the brain suggests that pathological αSyn retains the ability to propagate as prions. Transplant studies provided evidence that LB are present in normal fetal mesencephalic neurons grafted into the striatum of patients with PD, indicating that the pathology could be transmitted from the host brain to grafted healthy neurons [10, 11]. Moreover, inoculation of pathological brain homogenates from A53T mutant human αSyn transgenic (Tg) mice showing motor clinical abnormalities into A53T Tg mice [12–14] resulted in LB-like aggregations derived from endogenous αSyn and cell-to-cell spread, and the A53T Tg mice showed reduced survival period [12]. A similar prion-like phenomenon was also induced in WT mice inoculated with brain extracts from DLB patients [15] and A53T Tg mice inoculated with brain homogenates from multiple system atrophy (MSA) patients [14]. Furthermore, introduction of synthetic fibrils formed from r-αSyn elicited intercellular transmission of pathological αSyn in primary neurons [16] and WT mice [17], which developed dopamine neuron loss that resulted in motor deficits. These reports suggested that αSyn behaves like a prion.

Previously, we developed an in vitro amplification technique, designated “real-time quaking-induced conversion (RT-QUIC),” for detection of prions in tissue and body fluids [18]. This assay is based on the propensity of prions to act as seeds and replicate in an autocatalytic process that converts recombinant prion protein as a substrate into amyloid fibrils, which retain the prion infectivity and strain properties of the original prion [19]. The present study was performed to investigate whether prion-like conformational conversion of r-αSyn is induced by brain tissue from patients with DLB using the RT-QUIC assay. More recently, it has been shown that RT-QUIC using r-αSyn can detect abnormal αSyn in the brain and cerebrospinal fluid (CSF) from DLB and PD [20]. The RT-QUIC assay of CSF from DLB and PD showed 92 and 95% sensitivity, respectively, with an overall specificity of 100%. The study provides no data regarding the seeding activity and the form of abnormal αSyn. Furthermore, we performed quantitative analysis of abnormal αSyn seeding activity and examined the relationship between prion-like behavior and Ser129 phosphorylation or multimeric status of αSyn.

## Methods

### Patients

DLB brain tissues were obtained at autopsy from seven patients with histopathological confirmation of the clinical diagnosis. Of these subjects, six were classified as having diffuse neocortical DLB type (DN-DLB), and the remaining one case was limbic DLB type (Li-DLB) according to Braak staging. Brain tissues of prion disease were obtained at autopsy from three patients with sporadic CJD (sCJD) and one patient with GSS associated with a Pro to Leu mutation at codon 102 of *PRNP* (P102L). sCJD subtypes were diagnosed according to the genotype at codon 129 of the *PRNP* gene and the physicochemical properties of abnormal prion protein (PrP^Sc^). They included two cases of type 1, codon 129 MM (MM1), and one case of type 2, codon 129 MM (MM2). Brain tissues with AD were obtained at autopsy from two patients that had received a neuropathological diagnosis of the presence of neurofibrillary tangles and neuritic plaques. The brain specimens were pure forms of AD with little or no coexisting LBD. Brain tissues from schizophrenia and breast cancer patients without histopathological changes in the brain were used as non-degenerative cases. All samples were taken from the frontal cortex and stored at −80 °C.

### Recombinant Human α-Synuclein Expression and Purification

**Fig. 1:**
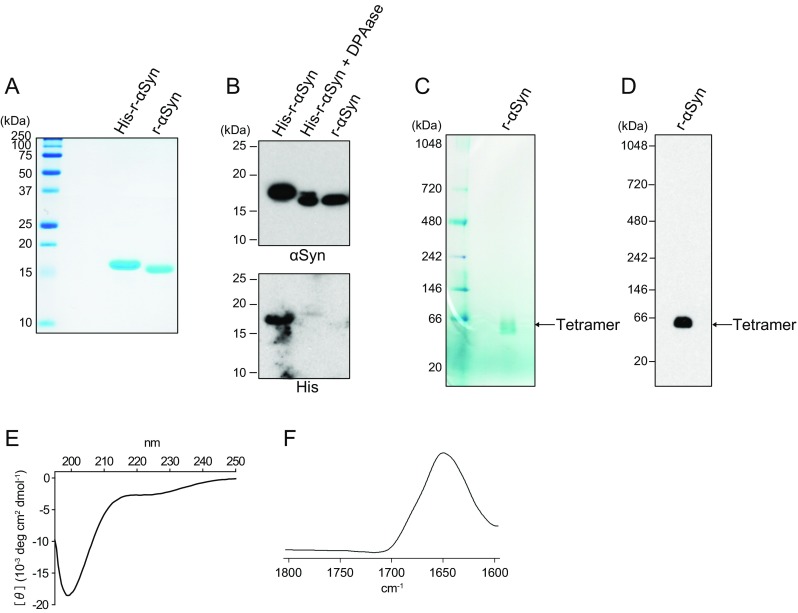
Purification and structural analysis of recombinant α-synuclein. **a**, **b** The purified His-tagged recombinant human αSyn (*His-r-αSyn*), His-r-αSyn after treatment with DAPase (*His-r-αSyn + DAPase*), and the product after removal of His-r-αSyn and DAPase (*r-αSyn*) were examined by Coomassie brilliant blue (*CBB*)-stained SDS-PAGE and western blotting analysis with polyclonal anti-αSyn antibody D119 and anti-His antibody. **a** CBB-stained SDS-PAGE analysis showed that the size of His-r-αSyn was shifted from 18 to 17 kDa after treatment with DAPase, and a single major band of the protein was observed after removal of His-r-αSyn and DAPase. **b** A similar shift in molecular size of His-r-αSyn was also observed by immunoblotting using polyclonal anti-αSyn antibody D119. The purified r-αSyn was confirmed not to be detected by immunoblotting using anti-His-tag antibody. Molecular mass markers are indicated in kilodaltons (*kDa*) on the left side of each panel. **c**, **d** r-αSyn was loaded onto 4% – 16% Bis-Tris native PAGE gels (Invitrogen) and separated by BN-PAGE. **c** CBB-stained BN-PAGE and **d** western blotting analysis with polyclonal anti-αSyn antibody D119 revealed a native αSyn band at approximately 60 kDa, suggesting a tetramer (theoretical mass of monomer  =  14,460.1 Da). **e** Circular dichroism (*CD*) spectrum of r-αSyn showed a minimum mean residue ellipticity at 199 nm, characteristic of disordered protein. **f** Fourier transform infrared spectroscopy (*FTIR*) spectrum of r-αSyn. The FTIR spectrum showed a prominent band at 1650 cm^−1^ assigned to a disordered structure

The DNA sequence encoding N-terminal His-tagged human wild-type αSyn residues 1 – 140 was amplified from human cDNA (Cat. No. FCC139B01; Toyobo) with forward primer (5′-ggaattccatatgaaacatcatcatcatcatcaccagatggatgtattcatgaaagg-3′) and reverse primer (5′-ctagctagctagttaggcttcaggttcgtagtctt-3′). The S129A mutant was amplified with forward primer (5′-ggaattccatatgaaacatcatcatcatcatcaccagatggatgtattcatgaaagg-3′) and reverse primer (5′-ctagctagctagttaggcttcaggttcgtagtcttgatacccttcctcagcaggc-3′). A stop point for dipeptidyl peptidase I (DAPase, Cat. No. 34362; Qiagen) that digests N-terminal His-tags was introduced into the protein sequence by inserting a glutamine codon (cag) into the expression construct. The amplified PCR fragment was inserted into *Nde*I and *Nhe*I sites of the pET11a vector (Cat. No. 69436-3; Novagen) and confirmed by sequence analysis. After transforming the plasmids into competent BL21 DE3 *E. coli* cells (Cat. No. DS250; BioDynamics Laboratory), recombinant protein was expressed at 37 °C for 16 h using MagicMedia *E. coli* Expression Medium (Cat. No. K6815; Invitrogen). The cells were pelleted by centrifugation at 3000 rpm for 15 min at 4 °C were suspended in CelLytic B (Cat. No. B7435; Sigma-Aldrich) in the presence of 1 g/ml lysozyme (Cat. No. 120-02674; Wako) and 500 U/mL benzonase nuclease (Cat. No. 70664-3; Novagen). The lysate was centrifuged at 10000 rpm for 30 min at 4 °C, and the supernatant was incubated with Ni-NTA Superflow resin (Cat. No. 30430; Qiagen) at room temperature for 30 min and then loaded onto a gravity flow column (Muromac mini-column; Muromachi Chemical Inc). The protein was eluted with a buffer containing 300 mM NaCl, 50 mM Tris-HCl (pH 8.0), and 250 mM imidazole and dialyzed against 10 mM phosphate buffer (pH 7.0) in cellulose dialysis tubing (Cat. No. 68035; ThermoFisher Scientific) at 4 °C overnight. As shown in Fig. 1a, b, removal of the tag from N-terminal His-tagged human α-synuclein was performed using the TAGZyme system (Cat. No. 34300; Qiagen). The purified His-r-αSyn contains a glutamine stop point for N-terminal exopeptidase DAPase between the His-tag sequence and the first amino acid of αSyn. The tag was cleaved by 40 mU DAPase in the presence of 2.4 U glutamine cyclotransferase (Qcyclase, Cat. No. 34342; Qiagen) at 4 °C overnight. The products were incubated with Ni-NTA resin at room temperature for 30 min and loaded onto a gravity flow column to remove the uncleaved His-tagged protein. The protein without His-tag was present in the flow-through fraction. The glutamine residue was converted in the presence of Qcyclase to pyroglutamate, which acts as a stop point for DAPase digestion. The pyroglutamate was eliminated through the action of 1.25 U of pyroglutamyl aminopeptidase (pGAPase, Cat. No. 34342; Qiagen) at 4 °C overnight, and then the products were loaded onto a gravity flow column after incubation with Ni-NTA resin at room temperature for 30 min. Human αSyn was obtained in the flow-through fraction. DAPase, Qcyclase, and pGAPase carry a His-tag at their C-termini and were therefore removed using Ni-NTA resin. The final protein was dialyzed against 10 mM sodium phosphate buffer (pH 7.0) in cellulose dialysis tubing at 4 °C overnight and filtered with a 0.2-μm syringe filter (Cat. No. SLLGH25; Millipore). The purity of the protein samples was ≥99.9%, as estimated by SDS-PAGE and immunoblotting, and analysis by nondenaturing Blue Native PAGE (BN-PAGE) revealed a native αSyn band at approximately 60 kDa (Fig. 1c, d), suggesting a tetramer (theoretical mass of monomer  =  14,460.1 Da). Previous studies have shown that soluble r-αSyn forms a stable tetramer [21], and endogenous αSyn from normal tissues occurs physiologically as a folded tetramer that resists aggregation [22]. The analysis by circular dichroism (CD) and Fourier transform infrared spectroscopy (FTIR) showed the disordered conformation of r-αSyn (Fig. 1e, f). After purification, aliquots of the proteins were stored at −80 °C until use.

### RT-QUIC Experiments

We prepared reaction mixtures in 96-well, optical, black-bottomed plates with a non-treated surface (Cat. No. 265301; Nunc) in a final total volume of 100 μl. The surface material was polystyrene. To avoid contamination, we prepared all materials inside a biological safety cabinet and used aerosol-resistant tips. The final concentrations of reaction buffer components were 50 mM HEPES (pH 7.5) and 10 μM Thioflavin T (ThT). The concentration of r-αSyn was 100 – 150 μg/ml, and only freshly thawed r-αSyn was used. Brain tissues (frontal cortex region) were homogenized at 10% (*w*/*v*) in ice-cold PBS supplemented with a protease inhibitor mixture (Cat. No. 11836170001; Roche) using a multi-bead shocker. After centrifugation at 2000  ×  *g* for 2 min, supernatants were collected and frozen at −80 °C until use. Brain homogenate was diluted with PBS prior to the reactions. r-αSyn, suspended in 95 μl of reaction buffer, was loaded into each well of a 96-well plate and mixed with 5 μl of brain homogenate. The 96-well plate was covered with sealing tape (Cat. No. 236366; Nunc) and incubated at 40 °C in a plate reader (Infinite M200 fluorescence plate reader; TECAN) with intermittent shaking, consisting of 40 s of circular shaking at the highest speed (432 rpm), no shaking for 20 s, and then a 2-min pause to measure the fluorescence. The fluorescence intensity on the bottom of the plate was read every 10 min to monitor the kinetics of amyloid formation using monochromators with excitation and emission wavelengths of 440 and 485 nm, respectively. Six replicates of each diluted brain homogenate sample were measured for 96 h. Each diluted sample of insoluble aggregates and oligomers of r-αSyn was assayed in three to four and three to six replicates for 72 h, respectively. The maximal fluorescence intensity in reactions with 5 × 10^−5^ and 5 × 10^−6^ dilutions of non-DLB cases and without seed was 97.3 ± 6.7 (means ± standard deviation) arbitrary units in total 90 wells within 96 h. In addition, none of the reactions reached a fluorescence intensity > 120 arbitrary units. Therefore, we designated positive reactions as those with fluorescence intensity > 120 arbitrary units and calculated the seeding dose giving a positive reaction in 50% of replicate reactions (SD_50_) using the Spearman–Kärber method as described previously [23]. We observed slight variations in the optimal r-αSyn concentration (100 – 150 μg/ml) between the lots of r-αSyn, but the final sensitivity for DN-DLB case #1, which was used as the standard for calculating sensitivity, was approximately the same (SD_50_ values of 10^7^ – 10^8^/g brain).

### Preparation of Insoluble Aggregates and Oligomers of r-αSyn for RT-QUIC Analysis

Wild-type or S129A r-αSyn (400 μg/ml) was incubated at 37 °C without agitation in the presence or absence of 4 U/μl of casein kinase 2 (CK2, Cat. No; P6010L, New England Biolabs Inc) or 200 μM ATP (Cat. No. A2383-1G; Sigma-Aldrich) in reaction buffer (20 mM Tris-HCl, pH 7.5, 50 mM KCl and 10 mM MgCl_2_). To obtain insoluble aggregates and oligomers of r-αSyn, the reactions were stopped after 264 and 5 h of incubation, respectively. After 264-h incubation, the mixture of insoluble r-αSyn aggregates was diluted in 50 mM HEPES buffer (pH 7.5) to a final concentration of 125 μg/ml r-αSyn and stored at −80 °C until use. After 5-h incubation, the mixture of r-αSyn oligomers was diluted in 50 mM HEPES buffer (pH 7.5) containing 10 μM ThT to a final concentration of 125 μg/ml r-αSyn and immediately subjected to RT-QUIC. The RT-QUIC products were stored at −80 °C until use.

### Western Blotting

Brain tissue (frontal cortex region) was lysed with Triton-deoxycholate (DOC) lysis buffer (50 mM Tris-HCl, pH 7.5, containing 150 mM NaCl, 0.5% Triton X-100, 0.5% sodium deoxycholate, 2 mM EDTA, and protease inhibitors) for 30 min at 4 °C. After 2 min of centrifugation at 2000 × *g*, the supernatant was collected, and its total protein concentration was measured using a bicinchoninic acid (BCA) protein assay kit (Cat. No. 23227; Pierce). Samples were boiled for 5 min at 95 °C with sodium dodecyl sulfate (SDS) loading buffer (62.5 mM Tris-HCl, pH 6.8, containing 5% 2-mercaptoethanol, 2% SDS, 5% sucrose, and 0.005% bromophenol blue) and subjected to SDS-polyacrylamide gel electrophoresis (SDS-PAGE). The proteins were transferred onto an Immobilon-P membrane (Cat. No. IPVH304F0; Millipore) in transfer buffer containing 15% methanol at 300 mA for 2 h; the membrane was blocked with 5% nonfat dry milk in TBST (10 mM Tris-HCl, pH 7.8, 100 mM NaCl, 0.1% Tween 20) for 2 h at 4 °C and reacted with antibodies against αSyn (D119, Cat. No. BS3429; Bioworld Technology, Inc., RRID: AB_1662955, 1:2500), Ser129-phosphorylated αSyn (EP1536Y, Cat. No. ab51253; Abcam, RRID: AB_869973, 1:2500), Ser87-phosphorylated αSyn (Cat. No. sc-19893-R; Santa Cruz Biotechnology, RRID: AB_2192817, 1:500) or His-tag (Cat. No. 34670; Qiagen, RRID: AB_2571551, 1:1000). Immunoreactive bands were visualized by HRP-conjugated goat anti-rabbit IgG antibody (Cat. No. 111-035-003; Jackson ImmunoResearch Labs, RRID: AB_2313567) or goat anti-mouse IgG antibody (Cat. No. 115-035-003; Jackson ImmunoResearch Labs, RRID: AB_10015289), using an enhanced chemiluminescence system (GE Healthcare Life Sciences).

### Transmission Electron Microscopy

Negative staining was performed on carbon supporting film grids, which were glow-discharged before staining. Aliquots of 5 μl of the samples were adsorbed onto the grids, and the residual solution was absorbed with filter paper. The grids were stained with 5 μl of freshly filtered stain (2% uranyl acetate). Once dry, the samples were viewed with a transmission electron microscope (JEM-1400PLUS; JEOL).

### CD

Circular dichroism (CD) spectra were measured with a JASCO J-820 spectropolarimeter (JASCO) using a quartz cell with a 1-mm path length. The CD spectrum was obtained by averaging four scans in the wavelength range of 195 – 250 nm. r-αSyn was dissolved in 20 mM sodium phosphate (pH 6.5) and 150 mM NaCl buffer. The concentration of r-αSyn was 300 μg/ml.

### FTIR

Fourier transform infrared spectroscopy (FTIR) spectra were measured with a Bruker Tensor 27 FTIR instrument (Bruker Optics) equipped with a mercuric cadmium telluride (MCT) detector cooled with liquid nitrogen. Aliquots of 20 μl of the sample were loaded into a BioATRcell II attenuated total reflectance-type reflectance unit. A total of 128 scans at 4-cm^−1^ resolution were collected for each sample under constant purging with nitrogen, corrected for water vapor, and the background spectra of the buffer were subtracted.

### Histopathology and Immunohistochemical Staining

The brain tissues were fixed in 20% neutral buffered formalin, and 8-μm paraffin sections were prepared on glass slides with a microtome. After deparaffinization and rehydration, the tissue sections were subjected to staining with hematoxylin and eosin, and immunohistochemical staining using anti-Ser129-phosphorylated αSyn antibody (Cat. No. 015-25191; Wako, RRID: AB_2537218, 1:3000). To enhance the immunogenicity, the sections were prepared by heating for 40 min at 98 °C prior to incubation with primary antibody. Primary antibody binding was detected by the labeled streptavidin-biotin method (DAKO). Peroxidase-conjugated streptavidin was visualized with 3′3-diaminobenzidine (Cat. No. 7411-49-6; Wako) as the chromogen. Immunostained sections were lightly counterstained with Mayer’s hematoxylin.

### Statistical Analysis

The values of maximal fluorescence intensity and lag phase, the time required to reach a fluorescence intensity >120 arbitrary units, in reactions with brain homogenate within 96 h, or in reactions with oligomers of r-αSyn within 72 h on RT-QUIC were extracted, and then compared between dilutions including controls (reactions without seed or with mock sample) in each individual case or sample. In addition, the values in reactions with oligomers of r-αSyn were compared between samples at the same dilutions. Data for maximal fluorescence intensities were analyzed by one-way ANOVA, followed by the Tukey–Kramer test. The data for lag phase were subjected to log-rank and Tukey–Kramer tests. *P*  <  0.05 or *P*  <  0.01 was taken to indicate statistical significance.

## Results

### Ser129-Phosphorylated α-Synuclein Is Abundant in the Brain from Dementia with Lewy Bodies

**Fig. 2:**
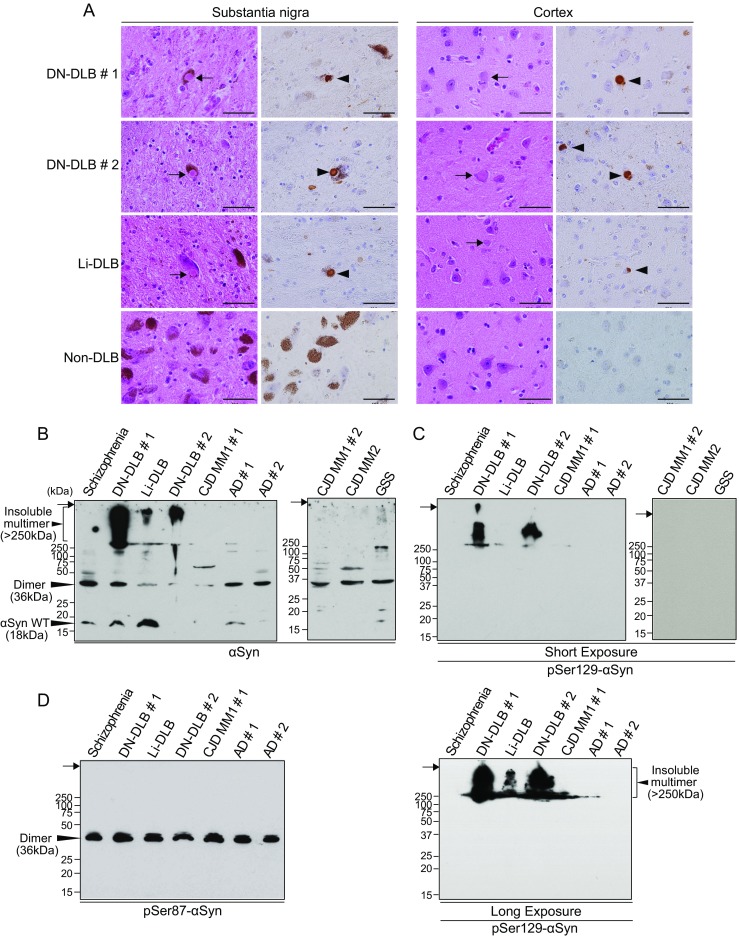
Characterization of α-synuclein from human brains by histochemical and western blotting analysis. **a** Hematoxylin and eosin staining and immunohistochemical staining using an antibody against phosphorylated αSyn in the substantia nigra and the cortex of the frontal lobe from two patients with DN-DLB (cases #1 and #2), a patient with Li-DLB, and non-DLB case (breast cancer patient). The *arrows* and *arrowheads* show LB and pSer129-αSyn, respectively. Scale bar, 50 mm. **b**, **c**, **d** The samples of BH from DN-DLB (cases #1 and #2), Li-DLB, schizophrenia, Alzheimer’s disease (*AD*) (cases #1 and #2), sporadic Creutzfeldt–Jakob disease (*sCJD*) type 1 (*sCJD MM1*) (case #1 and #2), sCJD type 2 (*sCJD MM2*), and Gerstmann–Sträussler–Scheinker syndrome (*GSS*) patients were immunoblotted with **b** polyclonal anti-αSyn antibody D119, **c** monoclonal anti-pSer129-αSyn antibody, and **d** polyclonal anti-Ser87-phosphorylated αSyn antibody. In the immunoblotting analysis for pSer129-αSyn presented in **c**, the samples were detected at a short exposure time of 30 s (*upper panel*) and long exposure time of 2 min (*lower panel*). Molecular mass markers are indicated in kilodaltons (*kDa*) on the left side of each panel. The *arrows* indicate the top of the stacking gel

First, we confirmed the presence of LB and αSyn phosphorylated at Ser129 (pSer129-αSyn) in the brains of DLB patients (Fig. 2a). Both LB and pSer129-αSyn were observed in sections of the substantia nigra and the cortex of the frontal lobe obtained at autopsy from two patients with diffuse neocortical DLB (DN-DLB) and one patient with limbic DLB (Li-DLB). In contrast, histochemical analyses revealed no pathological abnormalities in the brain of a non-DLB case. Consistent with a previous report [24], cells containing melanin granules in the substantia nigra were typically lost in patients with DN-DLB and Li-DLB, but not non-DLB, presumably due to neurodegeneration.

We next examined the biochemical properties of αSyn in brain homogenates (BH) from DLB patients by western blotting (Fig. 2b). Immunoblotting analysis of samples from all patients showed either a native αSyn band at 18 kDa, the dimeric form at 36 kDa, or both. The two cases of DN-DLB and one case of Li-DLB contained markedly bulky multimeric αSyn, which was in the mass range of > 250 kDa. The insoluble multimer was more abundant in DN-DLB than Li-DLB, indicating that αSyn multimer formation is critical to the disease progression. This band was not observed in non-DLB cases. All cases showed bands of various sizes in the mass range of around 50 – 250 kDa. These bands were probably due to the oligomeric and/or ubiquitinated αSyn, and did not differ significantly between cases. The antibody against pSer129-αSyn detected an apparent band at high molecular mass (> 250 kDa) in two cases of DN-DLB (Fig. 2c). A band of the same size was observed in one case of Li-DLB, but the intensity of immunoreactivity was much lower than in DN-DLB. In contrast, non-DLB cases showed no immunoreactivity to the antibody against pSer129-αSyn. These observations indicated the presence of an insoluble multimer of αSyn > 250 kDa in the DLB brain that was highly phosphorylated at Ser129. Furthermore, we estimated the levels of pSer129-αSyn in the brains of DLB cases by quantitative dot blot immunoassay following Phos-tag SDS-PAGE (Supplementary Fig. S1). The levels of pSer129-αSyn in DN-DLB cases #1 and #2 were 13.5  ±  0.4 and 3.7  ±  0.2 mg/g brain, respectively, and the level of Li-DLB was 0.06  ±  0.02 mg/g brain. The proportion of pSer129-αSyn to total αSyn in DN-DLB was higher than that in Li-DLB: DN-DLB case #1 (57.9%  ±  1.5%), DN-DLB #2 (22.6%  ±  1.2%), and Li-DLB (6.1%  ±  1.7%). These results suggest that pSer129-αSyn is relevant to disease progression. A previous study indicated that the level of αSyn phosphorylated on Ser87 is also increased in the LBD brain [25]. However, the antibody against Ser87-phosphorylated αSyn detected a dimeric band of 36 kDa, and there were no significant differences in levels of the Ser87-phosphorylated form between cases (Fig. 2d).

### Conversion of the Soluble Form of Recombinant Human α-Synuclein into Amyloid Fibrils by RT-QUIC

**Fig. 3:**
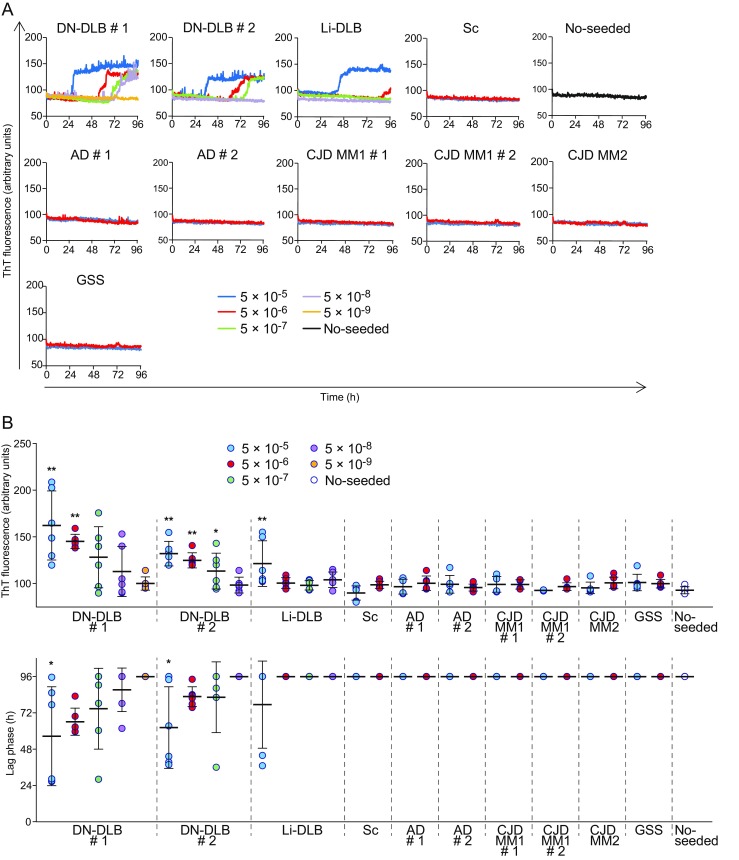
Fibril formation of r-αSyn in RT-QUIC reactions. **a** RT-QUIC was performed using human r-αSyn with the indicated dilutions of BH from DN-DLB (cases #1 and #2), Li-DLB, schizophrenia (*Sc*), AD (cases #1 and #2), sCJD MM1 (cases #1 and #2), sCJD MM2, and GSS patients, or without seed (no-seeded). The *colored curves* represent the kinetics of ThT fluorescence average of all six replicate wells. See also Supplementary Fig. S3. **b** The values of maximal fluorescence intensities and lag phase obtained in individual samples after 96-h reaction are plotted in the upper and lower graphs, respectively. Lag phase was defined as the time required to reach a fluorescence intensity > 120 arbitrary units. The *horizontal bars* indicate means  ±  standard deviation. The data for maximal fluorescence intensities were analyzed by one-way ANOVA, followed by the Tukey–Kramer test. Analysis of the data for lag phase was performed by the log-rank and Tukey–Kramer tests. **P  <  0.01 (compared with no-seeded); *P  <  0.05 (compared with no-seeded)

We next examined whether r-αSyn fibril formation could be induced in RT-QUIC by monitoring the levels of ThT fluorescence when BH from DLB patients was added to the reactions (Fig. 3a, b). When 5 × 10^−5^ and 5 × 10^−6^ dilutions of BH from two cases of DN-DLB were added, all reactions showed positive ThT fluorescence within 96 h. For the Li-DLB case, two of six reactions with a dilution of 5 × 10^−5^ showed positive responses. We also examined the effects of a dilution of 5 × 10^−4^ for Li-DLB, but no increase in fluorescence was observed (data not shown). The negative reaction was probably due to high levels of a variety of components in BH that inhibit r-αSyn fibril formation in the reactions. In contrast, unseeded controls and all reactions with dilutions of 5 × 10^−5^ and 5 × 10^−6^ schizophrenia BH produced no response over 96 h. The maximal fluorescence intensity was significantly higher in reactions with dilutions of 5 × 10^−5^ and 5 × 10^−6^ of DN-DLB case #1, dilutions ranging from 5 × 10^−5^ to 5 × 10^−7^ of DN-DLB case #2, and a dilution of 5 × 10^−5^ of Li-DLB compared to unseeded controls (Fig. 3b). The lag phase was significantly shorter in reactions with dilutions of 5 × 10^−5^ from two cases of DN-DLB compared to unseeded controls (Fig. 3b). The values of SD_50_/g brain of DN-DLB case #1 and #2 were 10^7.8^ and 10^7.3^, respectively. Although we could not calculate the seeding dose of Li-DLB accurately, the value was estimated to be less than 5.1 (log SD_50_/g brain) based on the assumption that the dilution of 5 × 10^−4^ showed 100% positivity. The specific detection of DLB was not observed in reactions with recombinant human prion protein as a substrate or protein-free reactions (Supplementary Fig. S2), indicating that DLB demonstrates the ability to seed on r-αSyn but not on other proteins. To further assess the influence of other protein misfolding and degenerative diseases on RT-QUIC, we applied BH from Alzheimer’s disease (AD), sporadic Creutzfeldt–Jakob disease (sCJD) type 1 and type 2, and GSS patients to the assay. In AD, sCJD (type 1 and type 2), and GSS, all reactions with dilutions of 5 × 10^−5^ and 5 × 10^−6^ gave negative responses within 96 h (Fig. 3a, b). These observations indicated that RT-QUIC induces the formation of r-αSyn fibrils only in the presence of BH from DLB cases, and that the seeding activity of DN-DLB is higher than that of Li-DLB. These findings suggest that r-αSyn can be converted into amyloid fibrils through a prion-like mechanism.

**Fig. 4:**
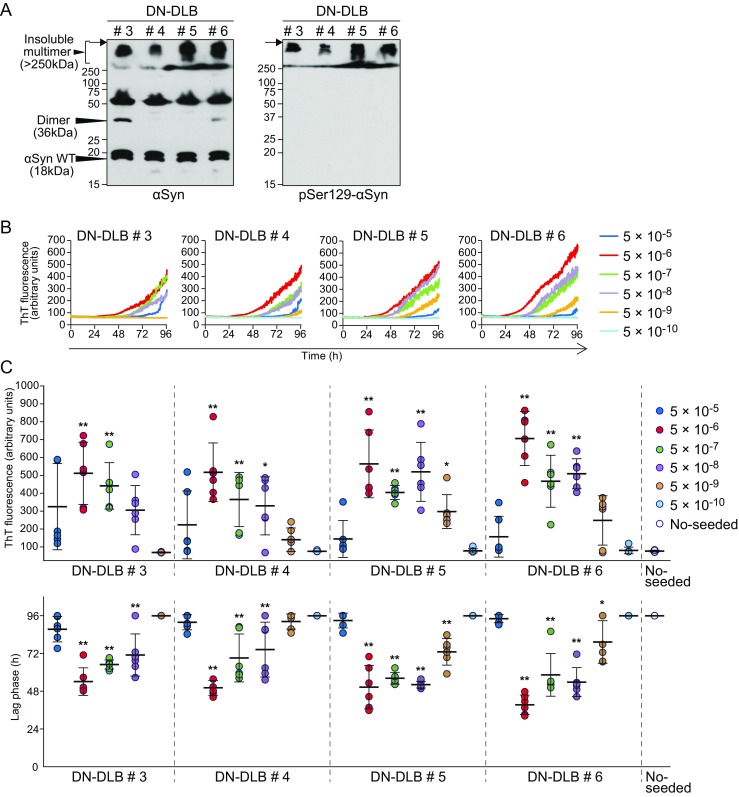
Western blotting and RT-QUIC analysis of another four DN-DLB brains. **a** The samples of BH from DN-DLB patients (cases #3, #4, #5 and #6) were immunoblotted with polyclonal anti-αSyn antibody D119 and monoclonal anti-pSer129-αSyn antibody. Molecular mass markers are indicated in kilodaltons (*kDa*) on the left side of each panel. The *arrows* indicate the top of the stacking gel. **b** RT-QUIC was performed using human r-αSyn with the indicated dilutions of BH from DN-DLB. The *colored curves* represent the kinetics of ThT fluorescence average of all six replicate wells. See also Supplementary Fig. S4. **c** The values of maximal fluorescence intensities and lag phase obtained in individual samples after 96-h reaction are plotted in the upper and lower graphs, respectively. Lag phase was defined as the time required to reach a fluorescence intensity > 120 arbitrary units. The *horizontal bars* indicate means  ±  standard deviation. The data for maximal fluorescence intensities were analyzed by one-way ANOVA, followed by the Tukey–Kramer test. Analysis of the data for lag phase was performed by the log-rank and Tukey–Kramer tests. **P  <  0.01 (compared with no-seeded); *P  <  0.05 (compared with no-seeded)

To further confirm the reliability of RT-QUIC, we analyzed BH samples from another four patients with DN-DLB (Fig. 4b, c). Similar to DN-DLB cases #1 and #2, immunoblotting analysis of samples from all patients showed an insoluble multimer of αSyn >250 kDa. The pSer129-αSyn was detected only in the multimer size (Fig. 4a). When 5 × 10^−6^ and 5 × 10^−7^ dilutions of these cases were added, all of the RT-QUIC reactions showed positive. Meanwhile, not all reactions with a dilution of 5 × 10^−5^ were positive, likely due to the presence of endogenous inhibitors of RT-QUIC reaction in BH. The maximal fluorescence intensity was significantly higher in reactions with dilutions from 5 × 10^−6^ and 5 × 10^−7^ in case #3, ranging from 5 × 10^−6^ to 5 × 10^−8^ in cases #4, and #6, and from 5 × 10^−6^ to 5 × 10^−9^ in case #5 compared to unseeded controls (Fig. 4c). The lag phase was significantly shorter in reactions with dilutions ranging from 5 × 10^−6^ to 5 × 10^−8^ of cases #3 and #4 and from 5 × 10^−6^ to 5 × 10^−9^ of cases #5 and #6 compared to unseeded controls (Fig. 4c). The values of SD_50_/g brain were as follows: 10^8.6^ (case #3), 10^9.3^ (case #4), 10^9.8^ (case #5), and 10^9.5^ (case #6). Thus, we were able to detect seeding activity of the brains from other DN-DLB patients. As shown in Fig. 3a, b, some of the reactions with DN-DLB #1 and 2 showed positive fluorescence around 24 h in contrast to the reactions with DN-DLB #3, 4, 5, and 6. Moreover, we observed differences in the shape of the fluorescence curve between both assays. We used different lots of r-αSyn for each assay, because the amounts of the lots were not sufficient. A preliminary study using different lots showed that the fluorescence curves were not necessarily similar in reactions with DN-DLB #1, and there are likely differences in fibril-forming properties, such as the rise time, the intensity of fluorescence, and the shape of the fluorescence curve, between lots of r-αSyn.

### Insoluble Aggregates of α-Synuclein Had Little or No Seeding Activity

**Fig. 5:**
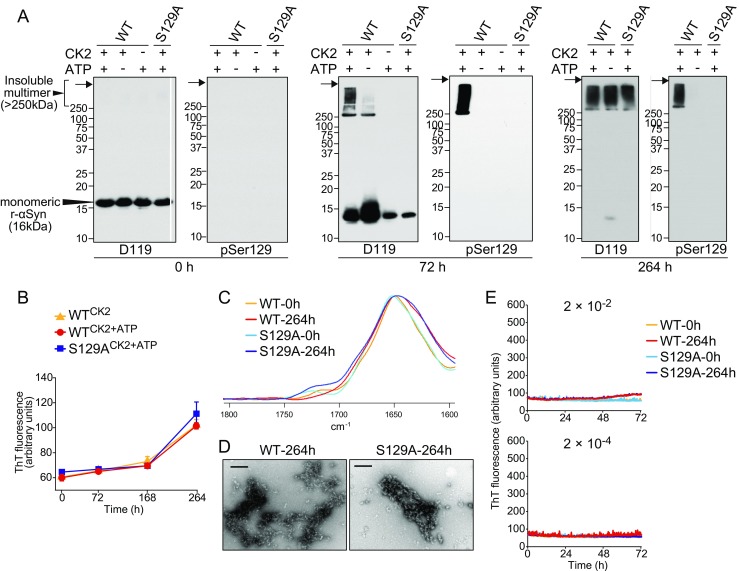
Seeding activity of insoluble aggregates of r-αSyn induced by incubation. WT or S129A r-αSyn (14 μg) was incubated at 37 °C in the presence (+) or absence (−) of 140 U of casein kinase 2 (CK2) or 200 μM ATP in 35 μl of reaction buffer (20 mM Tris-HCl, pH 7.5, 50 mM KCl, and 10 mM MgCl_2_). **a** After 0 (*left panel*), 72 (*central panel*), or 264 h (*right panel*) of incubation, the samples were immunoblotted with polyclonal anti-αSyn antibody D119 and monoclonal anti-pSer129-αSyn antibody. Molecular mass markers are indicated in kilodaltons (*kDa*) on the left side of each panel. The *arrows* indicate the top of the stacking gel. **b** Levels of ThT fluorescence of WT r-αSyn mixed with CK2 in the absence (WT^CK2^) or presence (WT^CK2+ATP^) of ATP and S129A r-αSyn mixed with CK2 and ATP (S129A^CK2+ATP^) were measured after 0, 72, 168, and 264 h of incubation. Data are expressed as means  ±  standard deviation (*n*  =  4). **c** WT or S129A r-αSyn incubated with CK2 and ATP for 0 (WT-0 h and S129A-0 h) or 264 h (WT-264 h and S129A-264 h) were subjected to FTIR analysis. **d** Samples were examined by TEM. Bars, 200 nm. **e** Seeding activity of WT-0 h, WT-264 h, S129A-0 h, and S129A-264 h samples was evaluated at dilutions of 2 × 10^−2^ and 2 × 10^−4^ by RT-QUIC. The *colored curves* represent the kinetics of ThT fluorescence averaged over three or four replicate wells

We next examined whether Ser129 phosphorylation is crucial for αSyn fibril formation through a prion-like mechanism using r-αSyn phosphorylated at Ser129 (pSer129-r-αSyn). WT r-αSyn was phosphorylated at Ser129 by incubation only in the presence of both casein kinase 2 (CK2) and ATP, whereas the S129A mutant was not phosphorylated under the same conditions (Fig. 5a). Similar to the case with DLB BH, the insoluble pSer129-r-αSyn was observed only in the mass range of > 250-kDa after 72- and 264-h incubation. Meanwhile, the insoluble aggregates of nonphosphorylated r-αSyn also converged on the mass range of > 250 kDa after 264-h incubation. Although there were no significant differences in increased levels of ThT fluorescence between WT r-αSyn incubated with CK2 in the absence (WT^CK2^) or presence (WT^CK2+ATP^) of ATP and S129A r-αSyn incubated with CK2 and ATP (S129A^CK2+ATP^) (Fig. 5b), the aggregate formation of pSer129-r-αSyn was induced more efficiently than that of nonphosphorylated r-αSyn after 72-h incubation (Fig. 5a). These results suggest that Ser129 phosphorylation accelerates polymerization of r-αSyn. Consistent with a previous report [26], a 13-kDa band with molecular weight lower than full-length r-αSyn was observed in all samples after 72-h incubation, indicating that r-αSyn aggregate formation is mediated by the truncation and/or degradation of r-αSyn. FTIR spectra of WT (WT-264 h) and mutant r-αSyn (S129A-264 h) incubated with CK2 and ATP for 264 h showed shifts to slightly lower wave numbers compared with before incubation (WT-0 h and S129A-0 h), indicating modestly increased β-sheet content (1630 to 1610 cm^−1^) (Fig. 5c). As shown in Supplementary Table 1, the β-sheet contents of WT-264 h (36.6%) and S129A-264 h (45.2%) were higher than those of WT-0 h (24.3%) and S129A-0 h (27.9%). There was little difference in the infrared spectra between WT and mutant r-αSyn before or after incubation (Fig. 5c). Transmission electron microscopy (TEM) analysis indicated that WT-264 h and S129A-264 h consisted exclusively of amorphous aggregates (Fig. 5d). We next examined whether r-αSyn could be newly converted into amyloid fibrils in the presence of amorphous r-αSyn aggregates in the RT-QUIC. Unexpectedly, all of the reactions with WT-264 h or S129A-264 h at dilutions of 2 × 10^−2^ and 2 × 10^−4^ yielded negative results in the RT-QUIC assay (Fig. 5e). Thus, the insoluble aggregates of r-αSyn had no prion-like seeding activity regardless of whether they were phosphorylated on Ser129 or nonphosphorylated.

**Table 1:** Assignments and relative proportions of secondary conformations of WT or S129A r-αSyn incubated with CK2 and ATP for 0 (WT-0 h and S129A-0 h) or 264 h (WT-264 h and S129A-264 h). The secondary structure content was determined by curve-fitting analysis of amide I components

WT-0 h		WT-264 h		S129A-0 h		S129A-264 h		
Peak (cm^−1^)	Area (%)	Peak (cm^−1^)	Area (%)	Peak (cm^−1^)	Area (%)	Peak (cm^−1^)	Area (%)	Assignment
1626	24.3	1625	36.6	1626	27.9	1629	45.2	β-Sheet
1642	30.2	1645	26.3	1643	29.0	1646	23.4	Disordered
1654	34.5	1658	29.8	1656	32.3	1659	23.4	α-Helix
1670	11.0	1678	7.3	1672	10.8	1681	8.0	β-Turn

### Oligomer-Like Forms of α-Synuclein Cause Prion-Like Propagation

**Fig. 6:**
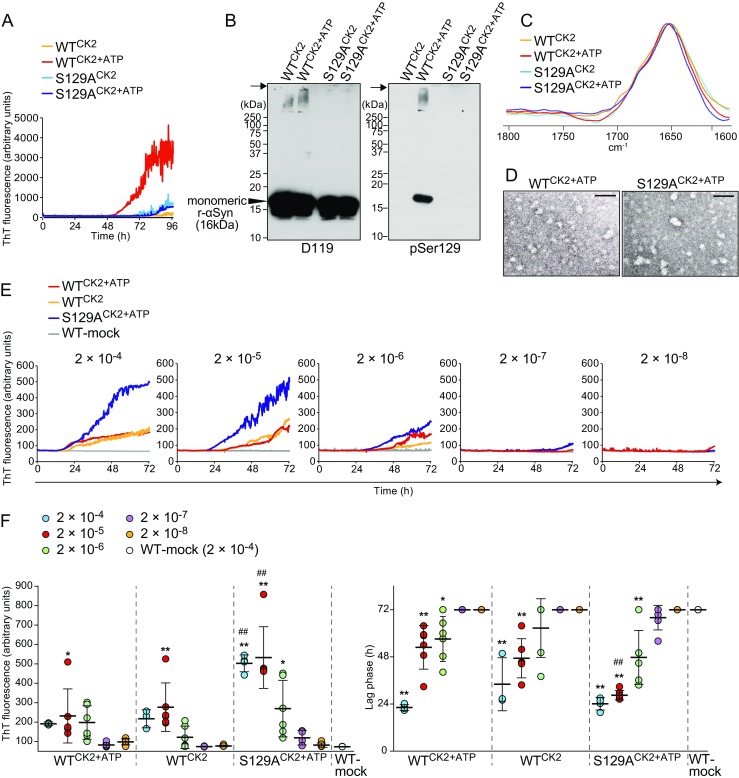
Seeding activity of r-αSyn oligomers generated by RT-QUIC. **a** Fibril formation of WT or S129A r-αSyn (12.5 μg) was induced in the presence of only 125 U of CK2 (WT^CK2^ and S129A^CK2^) or both 125 U of CK2 and 200 μM ATP (WT^CK2+ATP^ and S129A^CK2+ATP^) by RT-QUIC without seed. The *colored curves* represent the kinetics of ThT fluorescence averaged over triplicate wells. **b** RT-QUIC samples (WT^CK2^, WT^CK2+ATP^, S129A^CK2^, and S129A^CK2+ATP^) were immunoblotted with polyclonal anti-αSyn antibody D119 and monoclonal anti-pSer129-αSyn antibody. Molecular mass markers are indicated in kilodaltons (*kDa*) on the left side of each panel. The *arrows* indicate the top of the stacking gel. **c** RT-QUIC samples (WT^CK2^, WT^CK2+ATP^, S129A^CK2^, and S129A^CK2+ATP^) were subjected to FTIR analysis. **d** Samples were examined by TEM. Bars, 50 nm. **e** Seeding activity of RT-QUIC samples (WT^CK2^, WT^CK2+ATP^, S129A^CK2^, and S129A^CK2+ATP^) was evaluated at dilutions from 2 × 10^−4^ to 2 × 10^−8^ by subsequent testing by RT-QUIC. The *colored curves* represent the kinetics of ThT fluorescence averaged over replicate wells (*n*  =  3 – 6). **f** The values of maximal fluorescence intensities and lag phase obtained in individual samples after 72-h reaction are plotted on the left and right graphs, respectively. Lag phase was defined as the time required to reach a fluorescence intensity > 120 arbitrary units. The *horizontal bars* indicate means  ±  standard deviation. The data for maximal fluorescence intensities were analyzed by one-way ANOVA, followed by the Tukey–Kramer test. Analysis of the data for lag phase was performed by the log-rank and the Tukey–Kramer tests. **P  <  0.01 (compared with 2 × 10^−4^ dilution of WT-mock); *P  <  0.05 (compared with 2 × 10^−4^ dilution of WT-mock); ##*P*  <  0.01 (compared with the same dilution of WT^CK2+ATP^)

RT-QUIC using WT r-αSyn resulted in a more rapid increase and higher levels of fluorescence intensity in the reactions with CK2 in the presence of ATP (WT^CK2+ATP^) than in its absence (WT^CK2^), whereas there were no significant differences in ThT binding kinetics of S129A r-αSyn between the two conditions (S129A^CK2^ and S129A^CK2+ATP^) (Fig. 6a). The antibody against pSer129-αSyn detected a predominant band at 16 kDa and a weak band in the mass range of > 250 kDa only in WT^CK2+ATP^ (Fig. 6b). These results suggest that Ser129 phosphorylation accelerates fibril formation of r-αSyn in the RT-QUIC. Unlike the insoluble aggregates generated without shaking, as shown in Fig. 5a, all of the reactions predominantly showed a monomeric αSyn band at 16 kDa. In addition, the polymer larger than 250 kDa was barely detectable in WT^CK2+ATP^ and WT^CK2^. The difference in aggregation size of r-αSyn from Fig. 5a was probably due to shaking, which may cause the fragmentation of fibrils. FTIR analysis showed that there was little difference in the prominent band at 1650 cm^−1^ assigned to the disordered structure among all reactions (Fig. 6c). TEM analysis of WT^CK2+ATP^ and S129A^CK2+ATP^ revealed oligomer-like granular forms of r-αSyn (Fig. 6d). To examine whether these oligomer-like species show seeding activity, we performed a second passage of the RT-QUIC samples (Fig. 6e, f). The dilutions of 2 × 10^−4^ and 2 × 10^−5^ of WT^CK2+ATP^, WT^CK2^, and S129A^CK2+ATP^ showed 100% positivity in all of the reactions. The phosphorylated oligomer-like species yielded an SD_50_ value of 10^4.9^/μg r-αSyn (WT^CK2+ATP^), and the nonphosphorylated oligomer-like species showed SD_50_ values of 10^4.4^/μg r-αSyn (WT^CK2^) and 10^5.4^/μg r-αSyn (S129A^CK2+ATP^). In contrast, we observed no increase in fluorescence in any reactions with dilutions ranging from 2 × 10^−4^ to 2 × 10^−8^ of a mock sample (WT-mock) that contained the same components as WT^CK2+ATP^, which was prepared without shaking immediately before the assay. The maximal fluorescence intensity was significantly higher in reactions with WT^CK2+ATP^ and WT^CK2^ at a dilution of 2 × 10^−5^ and dilutions ranging from 2 × 10^−4^ to 2 × 10^−6^ of S129A^CK2+ATP^ compared to WT-mock (Fig. 6f). The lag phase was significantly shorter in reactions with dilutions ranging from 2 × 10^−4^ to 2 × 10^−6^ of WT^CK2+ATP^ and S129A^CK2+ATP^ and 2 × 10^−4^ and 2 × 10^−5^ dilutions of WT^CK2^ compared to WT-mock (Fig. 6f). Our results indicated that oligomer-like species of r-αSyn could display seeding activity with or without phosphorylation at Ser129.

## Discussion

The results of this study demonstrated that the formation of r-αSyn fibrils is induced by RT-QUIC using soluble r-αSyn only in the presence of BH from patients with DLB (Fig. 3a, b). The sodium dodecyl sulfate (SDS)-insoluble aggregates larger than 250 kDa detected only in DLB cases were specifically phosphorylated at Ser129 (Fig. 2c), and therefore we postulated that the insoluble aggregates of pSer129-αSyn confer prion-like seeding activity. Unexpectedly, however, the insoluble aggregates of r-αSyn with increased β-sheet structure had little seeding activity in both phosphorylated and nonphosphorylated states (Fig. 5e). Instead, the pre-fibrillar oligomers of r-αSyn exerted seeding activity both with and without phosphorylation (Fig. 6e, f). Our findings suggest that the soluble oligomeric, but not fully fibrillary, αSyn is a seeding species in vitro. Meanwhile, inoculation of fibrils generated from r-αSyn has been reported to induce prion-like spreading of pathological αSyn in WT mice [17]. The synthetic fibrils were formed with agitation (1000 rpm at 37 °C), and sonicated briefly prior to inoculation into mice, whereas the insoluble aggregates of r-αSyn used in Fig. 5e were generated by incubation at 37 °C without agitation. The fibril samples obtained using agitation and sonication are likely to contain oligomeric forms of r-αSyn, because these procedures are known to cause fibril fragmentation, resulting in shorter fibrils [27, 28]. Moreover, the fibrils may undergo partial proteolytic degradation in vivo. Therefore, the prion-like properties of synthetic r-αSyn fibrils reported in previous in vivo studies may be causally related to r-αSyn oligomers present in the fibril preparation and/or produced by degradation in vivo. Previous studies have shown that oligomeric species of r-αSyn are internalized by primary neurons and neuronal cell lines and induce endogenous αSyn aggregation [29, 30]. Furthermore, there is substantial evidence suggesting that oligomeric forms of αSyn are responsible for neuronal cell death and neurodegeneration [31, 32]. In a postmortem study, levels of soluble αSyn oligomers were significantly higher in the brains of patients with DLB than AD and controls, while there were no significant differences in levels of total αSyn between the three groups [33]. These reports support the suggestion that pre-fibrillar αSyn oligomers represent the pathogenic species with prion-like behavior in LBD rather than mature fibrils or amorphous aggregates. A similar approach using monomeric amyloid-β (Aβ) indicated that the protein misfolding cyclic amplification (PMCA) assay could detect seeding activity associated with Aβ oligomers present in CSF from patients with AD [34]. Brain tissue from DN-DLB had SD_50_ values of 10^3.4^ and 10^3.1^/μg total αSyn in cases #1 and #2, respectively, and 10^2.1^/μg total αSyn in Li-DLB (Fig. 3a, b), while the values of WT r-αSyn oligomers generated by RT-QUIC were 10^4.4^ – 10^4.9^/μg r-αSyn (Fig. 6e, f). It is unclear whether all of the r-αSyn in RT-QUIC reactions is present in oligomeric forms, but if so 3.2 – 10% and 1.6 – 5.0% of total αSyn are estimated to be oligomers in the brains of DN-DLB cases #1 and #2, respectively, and 0.2 – 0.5% in that of Li-DLB. Although the precise role of the LB insoluble aggregates remains unclear, cyto- and neuroprotective roles have been reported in studies using cell lines [35] and *Drosophila* [4]. The seeding activity of S129A oligomers was higher than that of WT oligomers regardless of phosphorylation state (Fig. 6f), and small amounts of the insoluble aggregates were detected in reactions containing WT oligomers but not S129A oligomers on western blotting analysis (Fig. 6b). These results suggest that the insoluble aggregates confer protection against prion-like propagation of αSyn.

Although there were no significant differences in seeding activity of r-αSyn oligomers between Ser129-phosphorylated and nonphosphorylated forms, we found that the phosphorylation of r-αSyn accelerates self-assembly (Fig. 5a and Fig. 6a) consistent with previous reports [1]. Previous studies showed that an increase in pSer129-αSyn level precedes the appearance of LB in the brains of patients with LBD [36] and is induced by oxidative stress, mitochondrial dysfunction [37], and proteasomal inhibition [38] observed in the LBD pathology. Furthermore, pSer129-αSyn has been reported to have a protective effect against neuronal dysfunction [5, 39]. These results suggest that pSer129-αSyn results from the protective mechanism against neuronal dysfunction. The pSer129-αSyn would then promote initiation of self-assembly into pre-fibrillar oligomers and mature fibrils.

Consistent with the previous report [20], RT-QUIC allowed the differentiation of DLB from other degenerative disorders, such as AD and prion diseases, as well as from non-degenerative cases, suggesting that r-αSyn is largely unaffected by the ability of other misfolded proteins, i.e., amyloid-β, tau, and PrP^Sc^, to induce heterologous cross-seeding. Although there have been reports of cross-seeding interaction between αSyn and other misfolded proteins in vitro and in vivo [40], the effect on RT-QUIC reaction appears to be negligible. The discrimination of DLB from other degenerative disorders raises the possibility of application of specific detection using RT-QUIC to differential diagnosis. The brains from patients with DN-DLB had an SD_50_ value of 10^7–10^/g brain, and that with Li-DLB was estimated to have an SD_50_ value of approximately 10^5.1^/g brain. Thus, it was suggested that RT-QUIC has a higher detection sensitivity for DLB compared with testing for pSer129-αSyn using western blotting or ELISA and provided more precise brain biopsy or autopsy diagnosis. Several studies using ELISA or bead-based flow cytometric assay investigated the levels of total αSyn in the CSF and blood of patients with DLB and other synucleinopathies, but the results have been inconclusive and contradictory [41]. Meanwhile, it was shown that the levels of soluble αSyn oligomers in CSF and blood of DLB and PD are elevated compared with those of AD and controls in ELISA [42, 43]. These reports support the suggestion that oligomeric forms of αSyn are significant and promising targets for diagnosis of LBD and suggest the diagnostic potential of RT-QUIC through seeding of oligomers from CSF and blood of patients with LBD. Thus, the use of RT-QUIC along with the existing ELISA specific for αSyn oligomers is likely to be particularly advantageous in the differential diagnosis of LBD. The sample size was a relatively small for comparison between the different types of DLB because Li-DLB was examined in only one case in this study. Further replication studies with larger samples are warranted to determine whether RT-QUIC is valuable for discrimination between the different types of LBD, as well as whether this assay can be used with body fluids or other tissues.

In this study, we demonstrated the feasibility of using RT-QUIC for laboratory detection of potential seeding species of αSyn. Our data provide further support for the suggestion that the oligomeric forms of αSyn are the seeding species that cause prion-like propagation and play a significant role in the pathogenesis of LBD. We believe that this new method represents a robust tool for clinical diagnosis, screening for potential drugs, and advances our understanding of the role of αSyn as a prion-like protein in LBD.

## Electronic supplementary material

ESM 1 (DOC 1401 kb).
