# Supplementary material for: Prion-Like Seeding of Misfolded α-Synuclein in the Brains of Dementia with Lewy Body Patients in RT-QUIC
Source: Mol Neurobiol. 2017 May 26;55(5):3916–30. doi: 10.1007/s12035-017-0624-1 (PMC5884914; doi:10.1007/s12035-017-0624-1)
Supplement: Supplementary file 1 — (DOC 1401 kb). [file 12035_2017_624_MOESM1_ESM.doc]

# Supplemental Information

# Prion-like seeding of misfolded α-synuclein in the brains of dementia with Lewy body patients in RT-QUIC

Kazunori Sano^1#^, Ryuichiro Atarashi^2^, Katsuya Satoh^3^, Daisuke Ishibashi^4^, Takehiro Nakagaki^4^, Yasushi Iwasaki^5^, Mari Yoshida^5^, Shigeo Murayama^6^, Kenichi Mishima^1^, Noriyuki Nishida^4^

^1^Department of Physiology and Pharmacology, Faculty of Pharmaceutical Sciences, Fukuoka University, Fukuoka 814-0180, Japan

^2^Division of Microbiology, Department of Infectious Diseases, Faculty of Medicine, University of Miyazaki, Miyazaki 889-1692, Japan

^3^Department of Locomotive Rehabilitation Science, Nagasaki University Graduate School of Biomedical Sciences, Nagasaki 852-8523, Japan

^4^Department of Molecular Microbiology and Immunology, Nagasaki University Graduate School of Biomedical Sciences, Nagasaki 852-8523, Japan

^5^Department of Neuropathology, Institute for Medical Science of Aging, Aichi Medical University, Aichi 480-1195, Japan

^6^Department of Neurology and Bioresource Center (Brain Bank for Aging Research), Tokyo Metropolitan Geriatric Hospital and Institute of Gerontology, Tokyo, 173-0015, Japan

## Fig. S1 Estimation of Ser129 phosphorylated α-synuclein concentration in DLB brain by immunoblotting following Phos-tag SDS-PAGE.


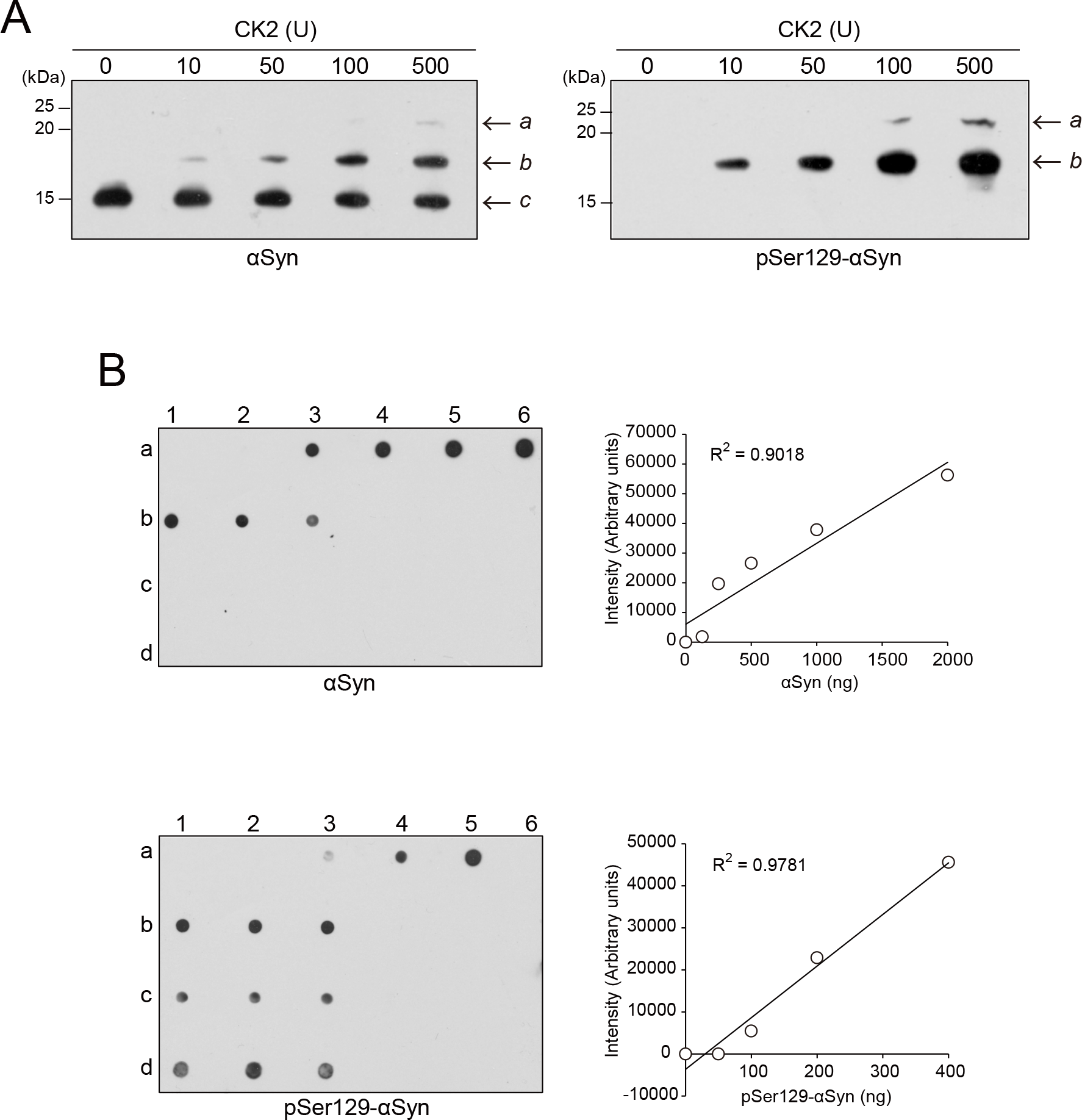


**a** To generate Ser129-phosphorylated r-αSyn (pSer129-r-αSyn) as standard for dot blotting analysis, r-αSyn was phosphorylated by incubation in the presence of casein kinase 2 (CK2) and ATP. The determination of pSer129-r-αSyn concentration was performed by Zn^2+^-Phos-tag SDS-PAGE followed by western blotting. Immunoblotting using anti-αSyn antibody D119 revealed that sample (1 μg of total r-αSyn) was separated into three different states (*a*, *b*, *c*) in a dose-dependent manner by CK2 (left panel). It should be noted that anti-pSer129 monoclonal antibody specifically recognized the (*a*) and (*b*) forms, indicating that the protein (*b*) is mono (Ser129)-phosphorylated r-αSyn and protein (*a*) is diphosphorylated at Ser129 and another amino acid (right panel). The pSer129-r-αSyn concentration in the sample treated with 500 U of CK2 was calculated to be 460 ng/μg of total r-αSyn from the ratio of the (*a*) and (*b*) bands (left panel). Although phos-tag SDS-PAGE is not dependent on the molecular weight, molecular mass markers were used as criteria for migration of proteins. Molecular mass markers are indicated in kilodaltons (kDa) on the left side of each panel. **b** The designated amounts of r-αSyn and pSer129-r-αSyn were used as standards for quantitative analysis of the extent of αSyn (upper left panel) and pSer129-αSyn (lower left panel) in BH, respectively. The code for standards of r-αSyn is as follows (upper left panel): a1, 0 ng; a2, 125 ng; a3, 250 ng; a4, 500 ng; a5, 1000 ng; a6, 2000 ng. The code for BH samples is as follows (upper left panel): b1, case 1 of DN-DLB (0.2% wt/vol); b2, case 2 of DN-DLB (0.2% wt/vol); b3, Li-DLB (2% wt/vol); b4, schizophrenia (2% wt/vol); b5, case 1 of CJD type 1 (2% wt/vol); b6, case 2 of CJD type 1 (2% wt/vol); c5, CJD type 2 (2% wt/vol); c6, GSS (2% wt/vol); d5, case 1 of AD (2% wt/vol); d6, case 2 of AD (2% wt/vol). The code for standards of pSer129-r-αSyn is as follows (lower left panel): a1, 0 ng; a2, 50 ng; a3, 100 ng; a4, 200 ng; a5, 400 ng. The code for BH samples is as follows (lower left panel): b1-3, case 1 of DN-DLB (0. 1% wt/vol); c1-3, case 2 of DN-DLB (0.2% wt/vol); d1-3, Li-DLB (20% wt/vol); b4, Schizophrenia (20% wt/vol); b5, case 1 of CJD type 1 (20% wt/vol); b6, case 2 of CJD type 1 (20% wt/vol); c5, CJD type 2 (20% wt/vol); c6, GSS (20% wt/vol); d5, case 1 of AD (20% wt/vol); d6, case 2 of AD (20% wt/vol). Samples of 20 μl were applied to nitrocellulose membranes (GE Healthcare Life Sciences). Linear regression between dot intensities and standards is shown in the upper right diagram for r-αSyn (r^2^ = 0.9018) and lower right diagram for pSer129-r-αSyn (r^2^ = 0.9781). αSyn accumulation was detected with anti-αSyn antibody D119 in BH from 2 cases of DN-DLB and one case of Li-DLB, whereas none was detected in non-DLB cases (upper panels). Immunoreactivity with the antibody against pSer129-αSyn were observed only in BH from two cases of DN-DLB and one case of Li-DLB (lower panels). The levels of pSer129-αSyn in DN-DLB cases #1 and #2 were estimated to be 13.5 ± 0.4 and 3.7 ± 0.2 mg/g brain, respectively, and the level of Li-DLB was 0.06 ± 0.02 mg/g brain. The rates of pSer129-αSyn to total αSyn were as follows: case 1 of DN-DLB (57.9 ± 1.5%), case 2 of DN-DLB (22.6 ± 1.2%), and Li-DLB (6.1 ± 1.7%).

**Fig. S2 RT-QUIC response in recombinant human prion protein-containing or substrate protein-free reaction buffer**

**
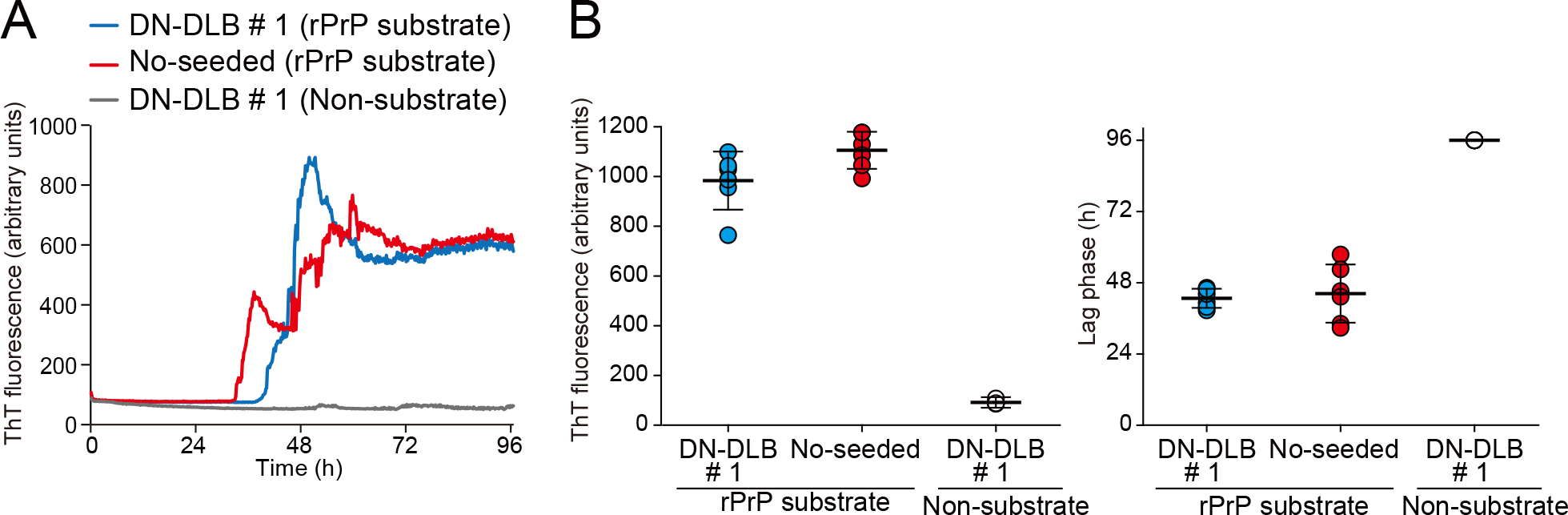
**

**a** RT-QUIC was performed in reaction buffer containing recombinant human prion protein (rPrP) with a dilution of 5×10^-6^ of DN-DLB case #1 or without seed (No-seeded), or in non-substrate protein containing reaction buffer with a dilution of 5×10^-6^ of DN-DLB case #1. The final concentrations of reaction buffer components were 50 mM HEPES (pH 7.5) and 10 μM Thioflavin T (ThT). The concentration of rPrP was 150 μg/ml. The colored curves represent the kinetics of ThT fluorescence average of all six replicate wells. **b** The values of maximal fluorescence intensities and lag phase obtained in individual samples after 96-hour reaction are plotted in the left and right graphs, respectively. Lag phase was defined as the time required to reach a fluorescence intensity > 120 arbitrary units. The horizontal bars indicate means ± standard deviation. There were no significant differences in the maximal fluorescence intensity or the lag phase in rPrP fibril formation between with and without seed. The non-substrate containing reactions resulted in no increase in ThT fluorescence.

## Fig. S3 Kinetics of r-αSyn fibril formation with seeds from DN-DLB (cases #1 and #2), Li-DLB, schizophrenia, AD, CJD and GSS


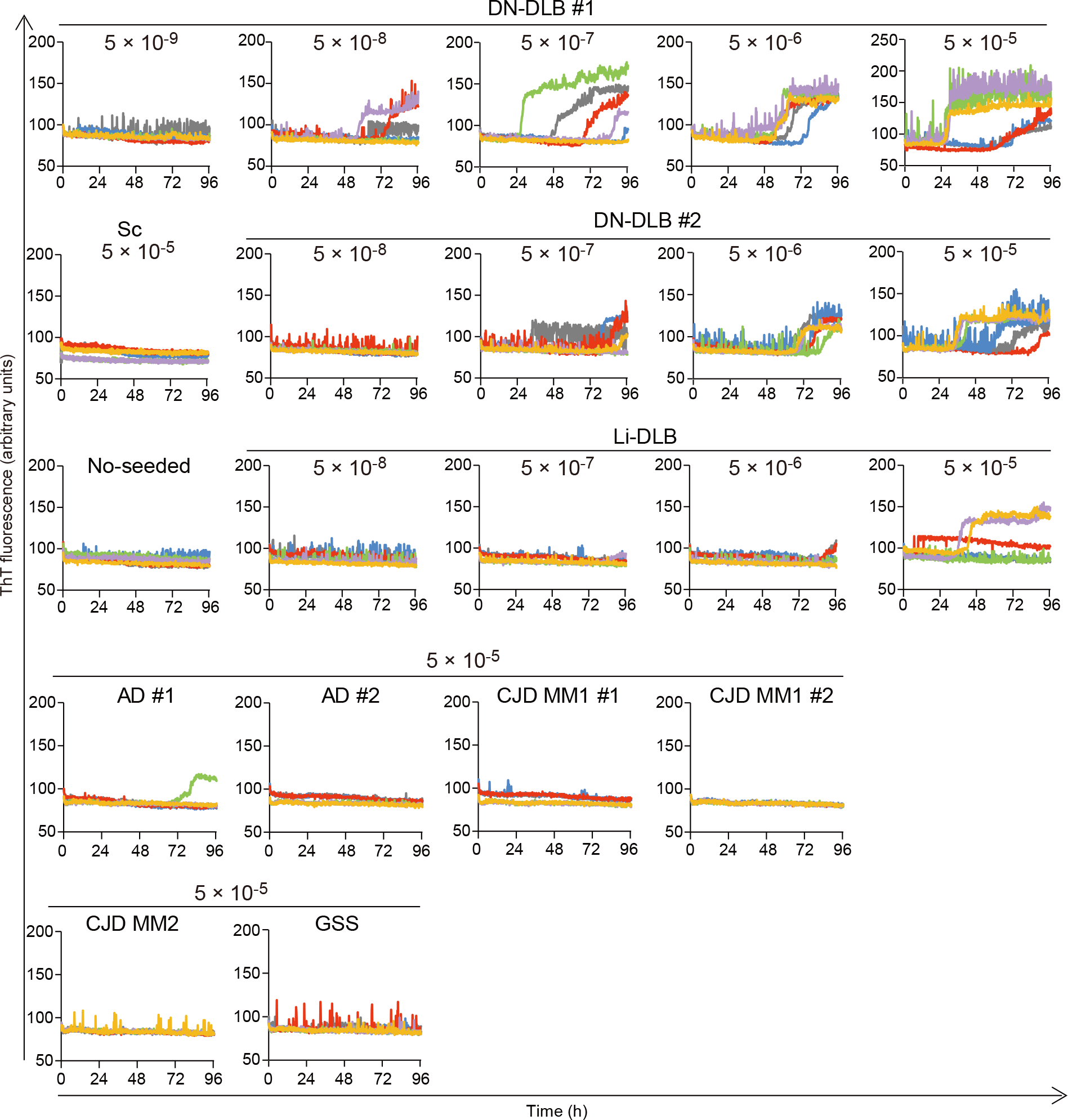


All reactions were performed in six replicates. The colored curves represent the kinetics of the ThT fluorescence from an individual reaction seeded with the same BH.

## Fig. S4 Kinetics of r-αSyn fibril formation with seeds from DN-DLB (cases #3, #4, #5 and #6)


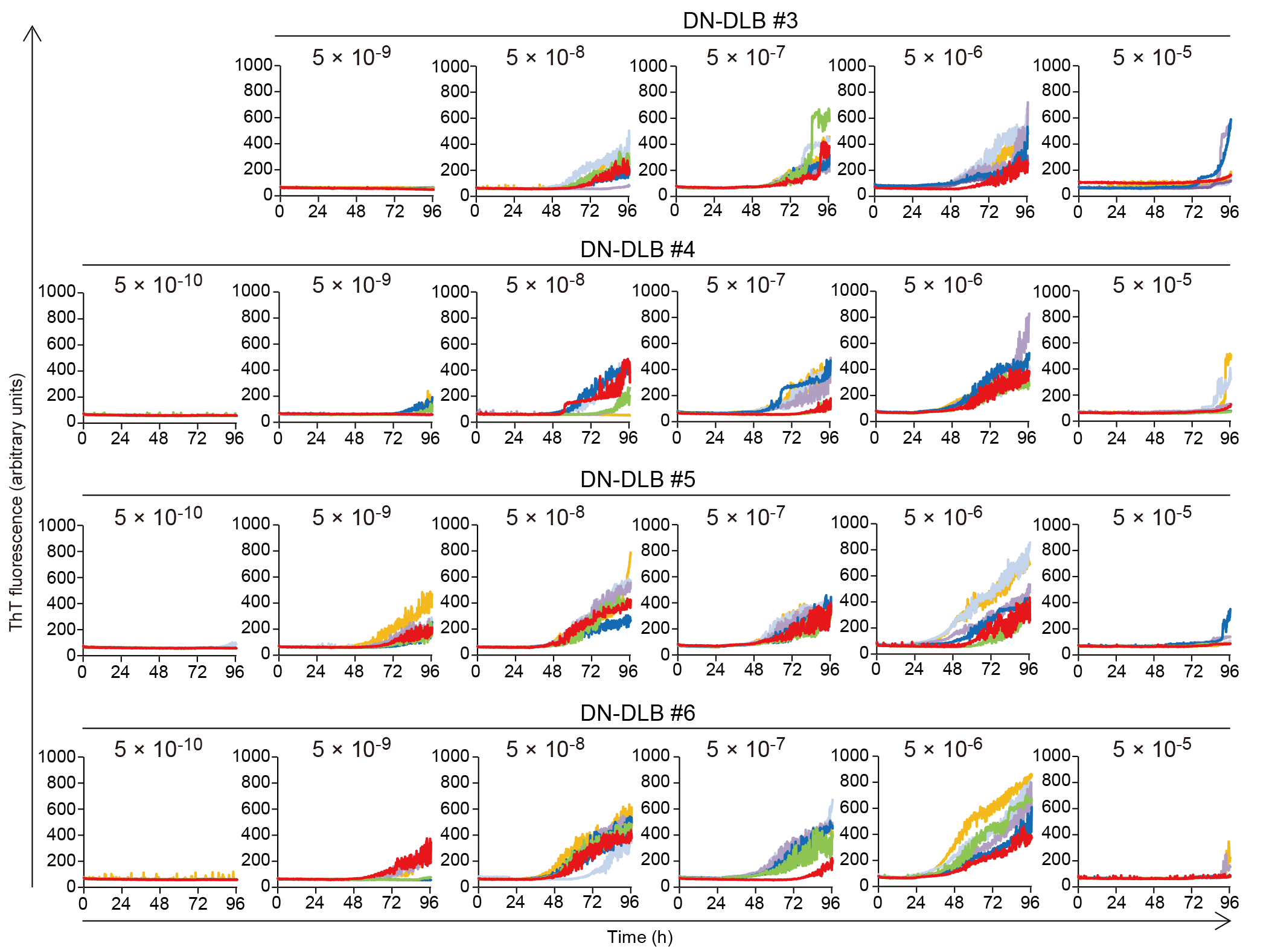


All reactions were performed in six replicates. The colored curves represent the kinetics of the ThT fluorescence from an individual reaction seeded with the same BH.

# Supplemental Experimental Procedures

## *In vitro* phosphorylation of recombinant α-synuclein

r-αSyn (3 μg) was incubated with casein kinase 2 (CK2, Cat. No; P6010L, New England Biolabs Inc) and 200 μM ATP (Cat. No. A2383-1G; Sigma-Aldrich) in 20 μl of reaction buffer (20 mM Tris-HCl, pH 7.5, 50 mM KCl, and 10 mM MgCl_2_) for 5 hours at 37°C. The reaction was stopped by boiling for 10 minutes at 95°C.

## Phos-tag SDS-PAGE and western blotting

Phos-tag SDS-PAGE was performed based on the tricine-SDS-PAGE method. Polyacrylamide running gels contained 1 M Tris-HCl (pH 8.45), 0.1% SDS, 13.3% glycerol, 100 μM Phos-tag, and 400 μM ZnCl_2_. The concentration of the anode buffer component was 200 mM Tris-HCl (pH 8.9). The concentrations of cathode buffer components were 100 mM Tris, 100 mM Tricine, and 0.1% SDS. Samples were boiled for 5 minutes at 95°C with SDS loading buffer (62.5 mM Tris-HCl, pH 6.8, containing 5% 2-mercaptoethanol, 2% SDS, 5% sucrose, and 0.005% bromophenol blue), and subjected to Phos-tag SDS-polyacrylamide gel electrophoresis. After electrophoresis, the gel was washed with transfer buffer containing 10 mM EDTA to chelate Zn^2+^ ions. The proteins were transferred onto an Immobilon-P membrane (Cat. No. IPVH304F0; Millipore) in transfer buffer containing 15% methanol at 300 mA for 2 hours. The membrane was blocked with 5% nonfat dry milk in TBST (10 mM Tris-HCl, pH 7.8, 100 mM NaCl, 0.1% Tween 20) for 2 hours at 4°C and reacted with diluted primary antibodies. Immunoreactive bands were visualized with HRP-conjugated secondary antibodies, using an enhanced chemiluminescence system (Amersham). Band intensities were determined using ImageJ 1.41.

## Dot blots

BH and r-αSyn were boiled for 10 minutes at 95°C with SDS loading buffer (62.5 mM Tris-HCl, pH 6.8, containing 5% 2-mercaptoethanol, and 2% SDS). The samples were blotted onto nitrocellulose membranes under mild vacuum-assisted conditions using a bio-blot (Bio-Rad, Hercules, CA, USA). After washing with TBST (10 mM Tris-HCl, pH 7.8, 100 mM NaCl, 0.1% Tween 20) and blocking with 5% nonfat dry milk in TBST for 2 hours, membranes were probed with diluted primary antibodies. Immunoreactive bands were visualized with HRP-conjugated secondary antibodies, using an enhanced chemiluminescence system (Amersham). Dot intensities were determined using ImageJ 1.41.
